# Role of Antioxidants in Cooled Liquid Storage of Mammal Spermatozoa

**DOI:** 10.3390/antiox10071096

**Published:** 2021-07-08

**Authors:** Miguel A. Silvestre, Jesús L. Yániz, Fernando J. Peña, Pilar Santolaria, María Castelló-Ruiz

**Affiliations:** 1 Departamento de Biología Celular, Biología Funcional y Antropología Física, Universitat de València, Burjassot, 46100 Valencia, Spain; Maria.Castello@uv.es; 2 BIOFITER Research Group, Institute of Environmental Sciences (IUCA), University of Zaragoza, 22071 Huesca, Spain; jyaniz@unizar.es (J.L.Y.); psantola@unizar.es (P.S.); 3 Laboratory of Equine Reproduction and Equine Spermatology, Veterinary Teaching Hospital, University of Extremadura, 10003 Cáceres, Spain; fjuanpvega@unex.es

**Keywords:** antioxidant, oxidative stress, refrigerated storage, semen, livestock species

## Abstract

Cooled preservation of semen is usually associated with artificial insemination and genetic improvement programs in livestock species. Several studies have reported an increase in reactive oxidative species and a decrease in antioxidant substances and sperm quality parameters during long-term semen storage at refrigerated temperatures. The supplementation of antioxidants in extenders before refrigeration could reduce this detrimental effect. Various antioxidants have been tested, both enzymatic, such as superoxide dismutase and catalase, and non-enzymatic, such as reduced glutathione, vitamins E and C and melatonin. However, the problem of oxidative stress in semen storage has not been fully resolved. The effects of antioxidants for semen-cooled storage have not been reviewed in depth. Therefore, the objective of the present study was to review the efficiency of the supplementation of antioxidants in the extender during cooled storage of semen in livestock species.

## 1. Introduction

Semen preservation, either by freezing or refrigeration allows the separation of the moment of extraction from that of use in artificial insemination (AI) or in vitro fertilization, providing multiple applications in livestock and human species [1,2]. In the case of livestock, semen preservation is usually associated with the AI technique and genetic improvement programs, allowing its use in places far from AI centers. AI is used for progeny testing of young males and for disseminating genetic improvement [3]. Cryopreservation or cooled liquid storage have different pros and cons [3], and the choice of the preservation method will depend on the AI efficiency in the specific species and the objective of the AI program. For example, frozen semen is usually used in bovine, while in porcine, AI is mainly performed with semen doses refrigerated at 15–18 °C and stored for several days. In general, fertility after AI is higher when using cooled rather than frozen/thawed semen.

In humans, spermatozoa are frozen to preserve fertility for the future (for example, prior to chemotherapy treatment [2]) or for depositing in donor banks. Sometimes cooled preservation can be useful for transporting raw semen samples from one laboratory or collection place to another for additional tests or uses [4]. Cooled semen is commonly used in domestic animals; therefore, the majority of the research studies concerning liquid cooled storage of semen referred to in this review were carried out with livestock species. Although freezing/thawing and refrigeration of semen are routine procedures in laboratories of livestock AI centers or human assisted reproduction clinics [1], these procedures are not always optimized, and a worsening of several important sperm quality parameters has frequently been observed [5,6].

Oxidative and nitrosative stress occurs when there is an excess of oxidants (reactive oxygen species (ROS) and reactive nitrogen species (RNS)), a deficiency of antioxidants, or both [7,8]. When sperm samples were stored cooled for a certain time, an increase in ROS [9,10,11] and a decrease in antioxidants [11] were observed. Treatments with antioxidants to avoid damage due to oxidative stress (OS) in gametes can be approached from different perspectives [12]. The first would be oral antioxidant supplementation, an approach widely discussed in different reviews on both male and female gametes in humans [6,13,14]. The second would be the supplementation of antioxidants in media used during assisted reproductive technologies, mainly in semen extenders used to preserve samples. In this context, the use of antioxidants in the frozen/thawing process has been extensively discussed in several works [15,16,17]. However, the effect of the inclusion of antioxidants in extenders used for semen cooled storage has not been reviewed in depth. Conclusions obtained in cryopreservation studies may not be applicable to refrigeration. There are substantial differences between the freezing and the refrigeration process, such as osmotic shock, cryoprotectant toxicity and/or the presence of ice crystals. In addition, cellular metabolism is practically stopped in frozen samples while in cooled storage sperm metabolism does not stop completely and the number of dead sperm progressively increases over time.

Therefore, the aim of this work was to review the efficiency of the use of antioxidants in the extender during liquid cooled storage of semen in mammals, mainly livestock species.

## 2. Oxidative/Nitrosative Stress in Spermatozoa

Oxidation occurs when an atom loses an electron. Free radicals (FR), including ROS and RNS, carry unpaired electrons and are normal pro-oxidant molecules [18,19]. The term ROS includes a set of oxygen metabolites, such as the radicals superoxide anion (O_2_^•—^) and hydroxyl (·OH), as well as the non-radical hydrogen peroxide (H_2_O_2_). The main ROS produced by spermatozoa is O_2_^•—^, mainly in two ways: (1) by the isoform 5 of the nicotinamide adenine dinucleotide phosphate oxidase (NOX5), which is a seven membrane-bound enzyme complex that catalyzes the reduction of oxygen to O_2_^•—^ using nicotinamide adenine dinucleotide phosphate hydrogen (NADPH) as an electron donor, producing ROS as its only function [20]; and (2) by aerobic metabolism at the mitochondrial electron transport chain level, essentially at complex I and III, due to the leakage of electrons from the electron transport chain that causes the partial reduction of oxygen to O_2_^•—^ instead of water [21,22]. This O_2_^•—^ could react with itself, through dismutation reactions, and generate H_2_O_2_, which is considered a ROS, due to its ability to react with Cu^2+^ and Fe^2+^ leading to the formation of ·OH via the Fenton reaction. H_2_O_2_ is a weak oxidant with important regulatory functions for cells [23]. In addition, mammalian spermatozoa also produce RNS [24], such as nitric oxide (·NO), a relatively unreactive FR [25,26]. Thus, ·NO can react with O_2_^•—^ generating the powerful and stable oxidant peroxynitrite (ONOO^−^). These two kinds of reactive species are often jointly named RONS. Spermatozoa are mitochondria rich cells because of their motility function, which causes a huge increase in their energetic demands. This fact may explain why the main source of RONS production in the spermatozoa is via oxidative phosphorylation. Furthermore, another two factors, leukocytes and immature or morphologically abnormal spermatozoa, present in ejaculates are involved in the genesis of oxidative/nitrosative stress. Spermatozoa together with the leukocytes present in the ejaculate are the major sources of ROS generation [27,28,29]. Leukocytes, mainly polymorphonuclears (neutrophils), are an important source of RONS in leukocytospermia situations (according to the WHO, when the number of white cells is greater than one million/mL in human semen [30]), with it being possible to establish a direct relationship between OS and an increased leukocyte count [31]. Additionally, immature spermatozoa present in the seminal fluid are another important source of RONS. Defects in the last phase of gamete differentiation may produce immature spermatozoa marked by an excess of cytoplasm in the mid-piece containing glucose-6P dehydrogenase, a pentose phosphate pathway enzyme, that controls the production of intracellular NADPH [13]. It has been demonstrated that high NADPH concentrations fuel the production of ROS via NOX5 [13,32]. Moreover, in several livestock species, dead spermatozoa may generate greater amounts of ROS [33] than live spermatozoa in the presence of phenylalanine substrate by the action of the L-amino acid oxidase (LAAO) enzyme [34,35]. This enzyme remains latent in live spermatozoa, but it is activated after the death of the sperm, probably due to an increase in membrane permeability [36]. Lastly, a further aspect which should be addressed is RONS diffusion capacity and lifetime. For example, because of its reactivity, cellular half-life of ·OH is approximately 10 s, but for H_2_O_2_ it is almost one minute. This means that, while ·OH can diffuse only a few angstroms from their source of production, H_2_O_2_ may diffuse several micrometers. In addition, not all RONS can cross the cellular membrane freely. Charged species like O_2_^•—^ cannot readily cross bilayers, while H_2_O_2_ is membrane permeable and could enter the cell more easily. These aspects could influence their ability to damage the living spermatozoa present in the ejaculate [37,38].

RONS play a dual role in mammalian spermatozoa, since they can be either physiologically required to achieve fertilizing ability [21,39], or be harmful, affecting their function and fertilizing potential [40,41,42]. At regulated and low concentrations (below the µM range), RONS play important roles in the physiological function of spermatozoa. A large number of studies demonstrate that controlled levels of RONS influence cellular signal transduction mechanisms which regulate sperm maturation, capacitation, hyperactivation, acrosomal reaction or sperm-oocyte fusion [21,35,43,44]. However, when RONS are produced in high concentrations, overpowering the antioxidant defense systems, they can lead the cell to a damaging state known as oxidative/nitrosative stress, in which the homeostatic balance between RONS production and antioxidant activity is broken. This state can have detrimental effects on sperm biomolecules such as lipids, nucleic acids or proteins which leads to alterations in the motility and fertilizing capacity of the sperm [45,46,47].

Paradoxically, despite the fact that sperm are considerable producers of RONS, they are one of the cell types most sensitive to them [48]. Lipid peroxidation (LPO) is a process in which FRs take electrons from the membrane lipids oxidizing them. This process often affects mostly polyunsaturated fatty acids (PUFA) due to the presence of double bounds near the methylene group, which weakens the methyl carbon-hydrogen bond, increasing the susceptibility of the hydrogen to oxidative damage. Mammalian sperm membranes are rich in PUFA [49,50] which, together with the low amounts of antioxidant enzymes in their scarce cytoplasm, makes sperm especially susceptible to injuries by RONS. The proportion of lipids in membranes varies between species, which could provide them with a higher or lower sensitivity to RONS [50]. Peroxidative damage to membrane lipids reduces sperm motility by reducing the membrane fluidity and altering the functions of membrane proteins involved in the maintenance of sperm motility [31], such as ATP-dependent ion pumps and voltage-regulated ion channels [51]. In addition, membrane LPO alters the fertilization process affecting the ability of spermatozoa to participate in membrane fusion events [52,53]. The LPO chain reactions also result in the formation of the end-products malondialdehyde (MDA) and 4-hydroxynoneal (4HNE). MDA is an important biomarker for measuring levels of peroxidative damage in the spermatozoa. 4HNE is a hydrophilic molecule and can diffuse from membranes to other subcellular compartments. This may lead to severe spermatozoa dysfunction, altering the protein structure as well as increasing ROS production in the mitochondria, which finally induces cytochrome c release, caspase activation, DNA fragmentation and, eventually, apoptosis [51,54].

Elevated RONS levels also affect the male reproductive functions by damaging proteins and nucleic acids. RONS cause protein permanent structural modifications through thiol oxidation, sulfonylation and tyrosine nitrosylation reactions. These structural protein alterations can modify the catalytic activity of glycolytic enzymes, Krebs cycle enzymes, protein complexes of the electron transfer chain and oxidative phosphorylation, leading the cell to a reduction in ATP production, energetic failure and mitochondrial dysfunction [13].

After spermiogenesis, spermatozoa have no effective response to repair damaged DNA, but the oocytes do [55]. Mature sperm DNA is quite resistant to oxidative damage due to the high degree of packaging conferred by positively charged molecules known as protamines, which replace nuclear histones during the differentiation process. However, in genome areas which are not strongly protaminated, or when inadequate protamination of the sperm chromatin occurs, the DNA becomes vulnerable to direct attack by FR. Guanine base is commonly affected by OS, generating the base adduct 8-hydroxy-2′-deoxyguanoside [31]. The DNA base oxidative lesions are potentially mutagenic due to the incomplete spermatozoa base excision repairing system, which conducts to the formation of abasic pairs [56]. In fact, some studies clearly associate infertility with deficient protamination [57,58]. Furthermore, RONS can also increase apoptosis leading to caspase-mediated enzymatic degradation of the DNA [42].

## 3. Semen Antioxidants

To prevent oxidative/nitrosative stress, organisms are provided with a set of endogenous and exogenous substances that function synergistically to neutralize RONS overproduction. An antioxidant is understood to be any substance capable of eliminating directly or indirectly ROS or derivatives, or restoring redox homeostasis [59]. In male reproduction research studies, antioxidants have been classified from different points of view, either according to their action mechanism [7,59], as enzymatic or non-enzymatic substances [29,60], as endogenous or exogenous [61], or according to their lipophilic or hydrophilic nature [13]. These substances include (1) non-enzymatic antioxidants, which are low-molecular-weight molecules that scavenge existing FRs, and include endogenous compounds such as glutathione, coenzyme Q10, amino acids, small peptides or the hormone melatonin, and exogenous compounds such as vitamin C, vitamin E, polyphenols and carotenoids, which scavenge existing FRs; (2) enzymatic antioxidants like superoxide dismutase (SOD), catalase (CAT), glutathione peroxidase (GPx), glutathione transferase (GST), peroxiredoxins (PRDXs), and thioredoxins (TRX) which catalyze the quenching reactions of FRs [62]; and (3) metal-binding proteins which sequester free iron and copper ions, limiting the formation of RONS [16,62,63,64,65,66,67]. In addition, cells have antioxidant-repairing systems, processes that remove oxidized biomolecules before they alter the cellular metabolism. The repair system’s intervention consists in the proteolysis of oxidized proteins to amino acids by proteases and their subsequent synthesis de novo, the removal of oxidatively damaged lipid components fundamentally by members of the phospholipase and glutathione peroxidase family, and the enzymatic reparation of the oxidatively damaged nucleic acids by both direct and excision repair mechanisms [61,66].

Antioxidants are present in both seminal plasma and spermatozoa, although different types can be found in each compartment [68]. These include, among others, SOD, CAT, GPx, melatonin, vitamins C, A and E, pyruvate, taurine, hypotaurine, and carnitine [68]. The greatest antioxidant capacity of ejaculates is in seminal plasma (SP), since the cytoplasm of spermatozoa is highly reduced [29,69]. Therefore, the majority of antioxidants, both enzymatic and non-enzymatic, are found in the SP and determine the total antioxidant capacity (T-AOC) [29,65]. SP is one of the corporal fluids with an estimated T-AOC ten times higher than blood [16]. However, this T-AOC is influenced by several factors as the inter-individual variability, male age and ejaculation frequency, and the season [65,68,70]. Moreover, its correlation with fertility or sperm quality parameters varies depending on the species. In humans and dogs, it has been observed that the T-AOC differs between fertile and infertile males and is related to several sperm quality parameters [71,72,73]. In bovine, there are differences between males, but not between ejaculates of the same male, and the T-AOC is not related with GPx or SOD content [70]. In porcine, no correlation was found between sperm motility and viability and T-AOC of SP after refrigerated storage [65]. However, the group of samples with low T-AOC of SP showed lower sperm motility and viability [65]. Several factors may influence the enzymatic antioxidant activity in semen. Amounts of SOD vary among mammal species, with the highest enzymatic activity being found in donkeys, rats and stallions [62]. Additionally, age could influence the enzymatic antioxidant content of SP. For example, a lower content in GPx and SOD was observed in older bulls [74]. In some species, like ovine or porcine, the activity of enzymatic antioxidant has been shown to vary according to the season [75,76]. Moreover, a large number of factors such as age, nutritional deficiencies or genetics could provoke antioxidant deficiencies [68]. These facts discussed above could explain, in part, the variability in sensitivity to ROS found in mammalian spermatozoa.

## 4. Sperm Cooled Storage and Oxidative Stress

During storage at refrigerated temperatures, usually from 17 °C to 4 °C, spermatozoa undergo changes that could compromise their fertilizing ability. Refrigerated liquid storage reduces sperm metabolism, but it does not completely arrest it [77]. At 5 °C, the Na^+^/K^+^ pump does not work adequately, increasing the Na^+^ intracellular concentration [78]. Moreover, during cooled storage, the cholesterol efflux increases, and capacitation or acrosome exocytosis may occur [79]. Furthermore, a weakening of the anchorage of proteins to the sperm membrane bilayer was observed during cooled storage, facilitating the loss of membrane integrity [80]. Previous to the reduction in temperature during the production of AI doses, SP is usually removed (washing process) by centrifugation or diluted to optimize the number of doses per ejaculate. The centrifugation process is related with sperm ROS formation, mainly due to the duration of centrifugation more than the g-force [42,69,81]. In addition to the centrifugation effect, the removal or dilution of the SP reduces the antioxidant capacity of samples still containing ROS-generating leukocytes. There are two options to reduce a possible OS: either the incorporation of antioxidants to the medium or the targeted elimination of leukocytes [16], although this latter is not common in domestic species.

In addition to the effect of cooling, centrifugation and elimination or dilution of SP antioxidants, the presence of dead sperm increases RONS by means of the LAAO enzyme and the presence of aromatic amino acids. Several authors attribute the decrease in sperm quality during cooled storage to the presence of dead sperm [78,82,83,84]. This could be especially relevant in prolonged liquid cooling storage when the dead sperm rate increases with storage time and when using extenders based on animal proteins such as egg yolk or milk [78], which are sources of phenylalanine, which is quite common. However, the presence of phenylalanine is not always detrimental. Recently, Castiglione-Morelli et al. [85] observed that a stallion group with high sperm motility had greater phenylalanine amounts in the SP, and those were correlated both at 0 h and after 24 h of liquid storage at 5 °C. In bulls, it has also been observed that phenylalanine was more abundant in “good freezers” and correlated with viability after thawing, postulating a possible antioxidant effect [86].

During long-term liquid cooled storage, various studies have reported an increase in ROS and MDA and a decrease in antioxidant substances [9,10,11,79,87,88] and other sperm quality parameters in different species, mainly total and progressive motility and membrane integrity (hypo-osmotic swelling test or viability) [3,11,89,90,91]. The addition of antioxidants can reduce this detrimental effect [9,87,89,92]. The significance of this detrimental effect of cold storage on sperm could vary depending on different variables [78]. One of the most important is the duration of cooled storage, as several authors did not find significant detrimental effects in the first hours of storage [3,78,80,93]. Additionally, the semen extender used during liquid storage may influence the results [9,84,94,95]. Very recently, it was observed that percentage of spermatozoa producing high amounts of ROS was reduced when they were cool stored in extenders with low glucose concentration [96]. Finally, as in cryopreservation, there would be males classified as “good coolers or poor coolers”, as mentioned by Aurich (2005) [97]. However, some males only maintained the classification of “good coolers” if SP was removed [98]. Analyzing proteome of equine SP, males classified as “poor coolers” showed a several SP proteins underrepresented in comparison with other males [98].

## 5. Treatments with Antioxidants in the Preservation Process

### 5.1. Enzymatic Antioxidants

Antioxidant enzymes present in spermatozoa and/or seminal plasma include SOD, CAT and GPx. As Table 1 shows, SOD and CAT are the most extensively studied enzymatic antioxidants. SOD is the main antioxidant enzyme in seminal plasma [15,99] and protects the cell against O_2_^•—^, as it catalyzes the dismutation of this anion to H_2_O_2_. Additionally, this reaction prevents the formation of the highly reactive ·OH which happens when O_2_^•—^ and H_2_O_2_ react with ferric ion by the Haber-Weiss reaction [100]. However, SOD activity promotes the formation of H_2_O_2_, a more stable and long-lived ROS, which can be removed by the cell using other enzymatic antioxidants such as CAT and GPx. In general, the addition of SOD to extenders, both alone or in combination with other antioxidants, has been found to increase sperm motility and viability in comparison with control groups in several species, although in canine and ovine these effects were not always evident (Table 1; [9,77,87,101,102,103,104]). In dogs, supplementation with SOD or SOD plus GPx did not improve the majority of sperm quality parameters in comparison with the control group [101].

The CAT enzyme catalyzes the reaction to convert H_2_O_2_ into water [61]. CAT was one of the first enzymatic antioxidants to be related to OS and sperm motility in McLeod’s work in 1943 [16], and it is the most extensively studied enzymatic antioxidant, alone or in combination, in sperm cooled storage in livestock species. As with SOD, a positive effect of CAT has also been observed in several species, mainly increasing sperm motility [77,79,105]. However, no effects, or even detrimental effects, were observed in equine [95,106].

The GPx enzyme exerts its antioxidant actions by using the reduced form of glutathione (GSH) as an electron donor to reduce H_2_O_2_ to water. Because of this reaction, GSH is oxidized to glutathione disulfide (GSSG), so finally another member of the GSH family of enzymes, glutathione reductase (GR), is responsible for regenerating GSH by transferring a proton from NADPH to GSSG [107]. Despite its importance in reducing H_2_O_2_, we found only one research study in the last 25 years using GPx alone in liquid cooled storage of spermatozoa [77], and in only a few studies has it been used in combination with other antioxidants in canine, equine and ovine [9,101,103,104]. Other studies on the effect of GPx on sperm quality parameters after freezing/thawing have been reported, but without conclusive results [108,109,110]. Antioxidant action of GPx is conditioned by the presence of GSH and H_2_O_2_ and this latter, in turn, is influenced by the presence of SOD. This is may be the reason that GPx has been studied more in combination with other antioxidant substances than alone.

**Table 1:** Effects of enzymatic antioxidants in liquid cooled storage on sperm parameters.

Antioxidant	Concentration	Opt	A/C	Temp	Time	Species	In Vitro Effects	Ref.
CAT	50–150 U/mL	100	A	4 °C	30 h	bovine	Increased sperm motility and decreased dead or abnormal spermatozoa, and acrosomal abnormalities compared with control group.	[79]
CAT	100 U/mL		A	4 °C	72 h	canine	Reduced total ROS, increased sperm motility.	[105]
CAT	90–3600 U/mL		A	5 °C	72 h	equine	No effect or detrimental effect at high concentrations.	[95]
CAT	100–200 U/mL		A	5 °C	72 h	equine	No effect on sperm motility. Increased viability in certain cases.	[106]
CAT	100–800 U/mL	100/200	A	5 °C	4 d	ovine	Increased sperm motility only on 4th day.	[77]
CAT	100–400 mM	200–400 mM	A	5 °C	24 h	ovine	Slight effects on sperm motility.	[111]
GPx	1–10 U/mL	10 U/mL	A	5 °C	6 d	ovine	Improved sperm motility on 6th day.	[77]
GPx	1–10 U/mL	10 U/mL	A	25 °C	6 d	ovine	Improved sperm motility on 6th day.	[77]
SOD	50–150 U/mL	100	A	4 °C	30 h	bovine	Increased motility and decreased dead or abnormal spermatozoa, and sperm acrosomal abnormalities compared with control group.	[87]
SOD	100 U/mL		A	4 °C	96 h	canine	No effect with respect to control group.	[101]
SOD	25–50 U/mL		A	5 °C	72 h	equine	Increased sperm motility and viability compared with control group.	[102]
SOD	100–800 U/mL	800 U/mL	A	5 °C	6 d	ovine	Improved sperm motility.	[77]
Mix: GSH + CAT	10 mM GSH + 100 IU/mL CAT		C	4 °C	72 h	ovine	No effect on viability or total motility. Reduced MDA.	[80]
Mix: Vit EP, SOD + Cat and GPx	Vit EP 12.5 μmol/L, SOD 37 μmol/L+ CAT 500 IU/mL, and GPx 20 IU/ml		C	5 °C	72 h	ovine	No effect on the studied sperm parameters (viability, acrosome).	[9]
SOD, CAT and GPx	15 IU/mL each of one		C	4 °C	10 d	canine	Increased total and progressive sperm motility, reduced DNA fragmentation mainly in hypofertile males.	[103]
SOD, CAT and GPx	15 IU/mL each of one		C	5 °C	72 h	equine	Increased motility and viability, and reduced DNA damage of spermatozoa compared with control group after 72 h storage.	[104]
SOD + GPx	100 and 5 U/mL respectively		C	4 °C	96 h	canine	No effect in sperm motility, DNA or acrosome status with compared with control group. Increased viability.	[101]

CAT: Catalase; SOD: superoxide dismutase; GPx: glutathione peroxidase; Mix: Enzyme and Non-Enzyme; Opt: optimum concentration; Time: time of cooled storage; A: Alone; C: Combination; d: days; h: hours; Temp: temperature; MDA: malondialdehyde; Ref.: reference.

### 5.2. Non-Enzymatic Antioxidants

#### 5.2.1. Amino Acids and Small Peptides

The antioxidant effect of many amino acids and small peptides, such as GSH, cysteine, hypotaurine, taurine, carnitine, glutamine, proline or methionine, has been studied in refrigerated semen samples (see Table 2). Thiols (−SH) such as cysteine, taurine, hypotaurine and GSH are a large class of antioxidants. Cysteine is, together with glutamate and glycine, one of the GSH components which supplies to this small peptide the –SH group, an essential chemical functional group for its scavenging actions. Among all of these, GSH is the most widely studied antioxidant in cooled semen, mainly in porcine, ovine and bovine. GSH is a low-molecular-weight compound made from the amino acids cysteine, glutamate and glycine. This small peptide exerts its antioxidant action in two ways: (1) directly neutralizing ROS, mainly due to the presence of the -SH deriving from the cysteine residue, and (2) maintaining other antioxidants such as vitamin C or E in their oxidized active forms. In addition, GSH protects cells by repairing damaged proteins, nucleic acids and peroxidated lipids, and maintaining a reducing state of the proteins’ sulphydryl groups [64]. Although numerous studies have been conducted using GSH as an antioxidant in semen extenders, the results still remain controversial. On the one hand, several studies found that supplementation of the medium with GSH increased motility, kinetics, viability and T-AOC [94,112,113,114,115,116,117]. However, many other studies found no effects or detrimental effects at high concentrations [94,111,112,118,119,120]. In rams in particular, the majority of studies found no effects of extender supplementation with GSH. It is possible that, in the case of GSH, the concentration may be determinant, and this may be variable depending on the species. In bovine and porcine, the optimal concentration seems to vary between 0.5 and 1.5 mM [94,112,114], but in ovine, the studied concentrations were much higher than in other species [115].

Another amino acid related to GSH used as an antioxidant in sperm cooled storage is cysteine. High levels of this amino acid are necessary to ensure adequate GSH levels, so under conditions of oxidative/nitrosative stress increased cysteine availability may be needed. When cysteine is oxidized, it is transformed to cystine in a reversible manner. Supplementation of cystine increased GSH and antioxidant capacity both in fresh and frozen/thawed spermatozoa [121]. Few studies have been conducted with different species using cysteine supplementation in extenders for cooled semen storage ([116,122,123]). Although in two of them, sperm motility and viability increased [116,123], these results are not conclusive.

Taurine, a sulphonyl amino acid derived from cysteine, and its intermediate hypotaurine have also been used as antioxidants in sperm extenders, mainly the former. In general, taurine reduced the drop of sperm motility, viability and acrosome integrity during cooled storage in several species. However, no beneficial effect was observed in ovine (Table 2; [91,105,119,124,125,126]).

Carnitine is a polar compound, highly distributed along the body and particularly concentrated in high energy demanding tissues such as the epididymis [127]. Since this compound has an important function transporting fatty acids into the sperm mitochondria, it plays a key role in sperm motility providing large amounts of energy through β-oxidation. In fact, increased motility of sperm in epididymal fluid has been related with the carnitine concentration [128]. However, carnitine is also an effective antioxidant, which (1) reduces lipid availability for peroxidation by allowing fatty acids to cross the mitochondrial membranes, (2) prevents OS protecting the antioxidant enzymes CAT, SOD and GPx from further peroxidative damage, and (3) has a direct scavenging action of FRs like O_2_^•—^ or H_2_O_2_ [129]. Carnitine has been used as an antioxidant in some studies in equine, porcine and rabbit, maintaining sperm quality parameters such as motility, viability and acrosome integrity during cooled storage (Table 2; [128,130,131,132]). The carnitine concentration used in these works was variable, even in the same species.

Finally, other amino acids such as glutamine and proline generally showed beneficial effects, increasing sperm motility, viability and reducing ROS during cooled storage compared to control groups (Table 2). In this regard, glutamine is an amino acid precursor of GSH, and proline has antioxidant properties based on its secondary amine structure [133]. Methionine is an amino acid capable of protecting cells from oxidative damage by acting as a precursor amino acid for cysteine, and also due to its capacity to react with oxidants to form methionine sulfoxide [134]. Several studies have been developed to evaluate the effect of glutamine, with these showing beneficial effects (Table 2; [135,136,137]).

**Table 2:** Effects of non-enzymatic antioxidants (amino acids and small peptides) supplementation in liquid cooled storage on sperm quality parameters.

Antioxidant	Conc	Opt	Temp	Time	Species	In Vitro Effects	Ref.
cysteine	5 mM		5 °C	72 h	caprine	No effect on sperm motility and HOST.	[122]
cysteine	2–4 mM		5 °C	96 h	ovine	Slightly increased motility and viability.	[123]
cysteine	0.25–5 mM	5 mM	10 °C	7–14 d	porcine	Increased sperm viability.	[116]
hypotaurine	5 mM		10 °C	7–14 d	porcine	No effect on viability.	[116]
arginine	4–6 mM	4 mM	5 °C	5 d	caprine	Increased total motility, viability and reduced MDA.	[138]
carnitine	50 mM	50 mM	20–25 °C	72 h	equine	Increased total motility and reduced ROS and lipid peroxidation.	[132]
carnitine	0.5–2 mM		5 °C	72 h	equine	Increased sperm motility. No effect on ROS or viability.	[130]
carnitine	12.5–100 mM	50 mM	17 °C	5–10 d	porcine	Increased motility, viability, acrosome integrity, mitochondrial activity and T-AOC. Reduced MDA and ROS.	[128]
carnitine	0.5–2 mM	2 mM	5 °C	24 h	rabbit	Increased total motility, viability and acrosomal abnormality.	[131]
glutamine	20–60 mM	60 mM	22 °C (24 h)/5 °C	72 h	equine	Increased total and progressive motility. No effect or detrimental effect on sperm viability.	[126]
glutamine	10–80 mM	20 mM	17 °C	5 d	porcine	Increased motility, velocities, viability and T-AOC. Reduced ROS production. Toxic at high concentrations.	[137]
glutamine	0.5–2 mM	1–2 mM	5 °C	24 h	rabbit	Increased total motility, viability and acrosomal abnormality.	[131]
Methionine	2–4 mM		5 °C	96 h	ovine	Increased motility and viability.	[135]
Methionine	1–12 mM		5 °C	96 h	Rabbit	No effect on studied parameters.	[136]
Methionine	1–12 mM		15 °C	96 h	Rabbit	No effect on studied parameters.	[136]
proline	20–60 mM	60 mM	22 °C (24 h)/5 °C	72 h	equine	Increased total and progressive motility. No effect on viability.	[126]
proline	25–125 mM	75	17 °C	5 d	porcine	Increased total and progressive motility, GSH levels and activities of CAT and SOD. Improved viability, MMP and ATP levels. Reduced ROS.	[133]
GSH	5–10 mM	5 mM	5 °C	48 h	equine	Increased total motility and viability, reduced MDA. Toxic at high concentrations.	[117]
GSH	0.5–3.0 mM	0.5 mM	4–8 °C	5 d	bovine	Increased motility. Reduced acrosomal damage. Toxic at high concentrations.	[112]
GSH	0.2–5 mM	1–1.5 mM	25 °C	24 h	bovine	Slightly improved progressive motility (depending on extender).	[94]
GSH	0.2–5 mM		5 °C	24 h	bovine	No effect on studied parameters.	[94]
GSH	1 mM		5 °C	72 h	caprine	Increased progressive motility and viability. Reduced lipid peroxidation.	[113]
GSH	0.2–5 mM		5 °C/15 °C	96 h	ovine	None or detrimental effect on motility and viability (improved mitochondrial activity at 5 °C) (depending on extender).	[118]
GSH	50–200 mM	200 mM	5 °C	72 h	ovine	Increased motility and kinetics, viability, T-AOC, and MMP.	[115]
GSH	100–400 mM		5 °C	24 h	ovine	Detrimental effects at high concentration.	[111]
GSH	5–10 mM	5–10 mM	5 °C	30 h	ovine	No effect on motility, increased viability.	[119]
GSH	5 mM		10 °C	7–14 d	porcine	Increased viability at 14 d	[116]
GSH	1–15 mmol/L	1 mM	17 °C	5 d	porcine	Increased motility, viability, T-AOC.	[114]
GSH	0.5–1.5 mM		5 °C	24 h	tigrina	No significant effect on studied parameters.	[120]
Taurine	20–60 mM	60 mM	22 °C (24 h)/5 °C	72 h	equine	Increased total and progressive motility. No effect on viability.	[126]
Taurine	25–100 mM	50 mM	5 °C	No data	bovine	Improved sperm motility, viability, acrosome integrity.	[124]
Taurine	0.2 mM		4 °C	72 h	canine	Increased motility and viability.	[105]
Taurine	100 mM		5 °C	5 d	equine	Increased total motility.	[125]
Taurine	50–100 mM		5 °C	30 h	ovine	No effect on motility or viability.	[119]
Taurine	0.5–10 mmol/L	5 mM	17 °C	72 h	porcine	Increased motility, viability, acrosome integrity and T-AOC. Reduced MDA.	[91]

GSH: reduced glutathione; SOD: superoxide dismutase; CAT: catalase; MMP: mitochondrial membrane potential; ROS: reactive oxygen species; HOST: hypo-osmotic swelling test; Opt: optimum concentration; Time: time of cooled storage; d: days; h: hours; Temp: temperature; T-AOC: Total antioxidant capacity; MDA: malondialdehyde; Ref.: reference.

#### 5.2.2. Vitamins, Carotenoids and Polyphenols

Vitamin E, vitamin C, polyphenols and carotenoids are all well-known natural antioxidants [139] which have been used to supplement semen extenders to palliate the detrimental effect of cooled storage (Table 3). The term vitamin E refers to a set of tocopherols (α, β, γ, δ) and tocotrienols (α, β, γ, δ). Among them, α-tocopherol is the most potent lipid-soluble antioxidant, and can block the LPO reaction chain by donating an electron to a lipid- or a lipid hydroperoxide radical, transforming itself into the relatively stable tocopheroxyl radical. The latter can be transformed back to the active tocopherol form by reacting with other antioxidants such as vitamin C or GSH [6,13]. Trolox is a synthetic water-soluble vitamin E analogue. The effect of vitamin E, both as α-tocopherol or Trolox, has been extensively studied in cooled semen in several livestock species (Table 3; [105,106,113,118,140,141,142]). However, most studies did not find a beneficial effect on sperm quality parameters.

Vitamin C is a hydrosoluble antioxidant due to its ability to function as a reducing agent, which can donate one or two electrons and oxidize itself to the ascorbyl radical or dehydroascorbic acid (DHA), respectively. Later, DHA can be reconverted back to the reduced form at the expense of GSH oxidation to GSSG. Antioxidant actions of vitamin C include both a direct scavenging action of a wide range of RONS including hydroxyl, superoxide and peroxynitrite radicals, and an indirect scavenging action of lipophilic radicals by reducing the tocopheroxyl radical to its active form tocopherol [155]. As with vitamin E, the supplementation of vitamin C to the semen extender did not exert a significant beneficial effect on sperm quality parameters during liquid cooled storage (Table 3; [94,105,106]). Only Aurich et al. [95] found vitamin C supplementation increased sperm viability, although it was toxic at high concentrations.

Lastly, polyphenols such as resveratrol, quercetin, procyanidin, hydroxytyrosol and 3,4-dihydroxyphenylglycol have been used as antioxidants in semen cooled storage (Table 3; [88,136,149,150,151,152,153,154]. Polyphenols are secondary plant-derived metabolites characterized by multiple phenol units. These compounds are structurally very diverse and include four principal classes: phenolic acids, flavonoids (such as quercetin), stilbenes (such as resveratrol) and lignans. Because of their chemical structure, these compounds are natural antioxidants that tend to oxidation, which allows them to intercept FRs and protect cells from oxidative damage. Moreover, some polyphenols have an enzymatic antioxidative action since they can upregulate antioxidant enzymes [6,156]. Among the numerous polyphenols studied, resveratrol has been the most frequently used, mainly in porcine semen [150,151,152,153,154]. However, the results are not conclusive, with more studies showing no effects than beneficial effects. It seems that the resveratrol concentration may be important (optimal concentration around 50 µM), since several studies have observed detrimental effects at high concentrations [150,152,153].

#### 5.2.3. Other Antioxidant Substances

Some hormones, including melatonin, show antioxidant properties [157]. Melatonin is a tryptophan-derived indole synthesized and secreted by the pineal gland during the night. In addition to its role in the regulation of the circadian cycle or seasonal reproduction in mammals, this hormone has significant antioxidant functions. Receptors of melatonin have been found in human, hamster and ram spermatozoa, in addition to their presence in the seminal fluid [158,159,160], but not in stallion sperm [161]. Melatonin has demonstrated both a direct antioxidant action scavenging some RONS, such as ·OH, O_2_^•−^, ONOO^−^ and ·NO [162], and an indirect antioxidant action by stimulating the activity of endogenous antioxidants such as CAT, SOD or GPx [157,163]. Melatonin has been used as an antioxidant in extenders in several studies in bovine, ovine, porcine, rabbit and equine, with a predominant increase in sperm motility, viability and reduced LPO with respect to control groups (Table 3 [83,93,143,144,145,146,147,148]). Semen incubated with melatonin at 37 °C maintained or improved quality sperm parameters and increased blastocyst rate after in vitro fertilization respect to control group [163]. Moreover, melatonin reduced detrimental effects of H_2_O_2_ on sperm parameters and in vitro embryo production [163].

Finally, Lycopene, a red carotenoid found in fruits and vegetables such as tomatoes, carrots or grapefruits, has been described as a potent antioxidant with an efficacy two times superior to that of β-carotene and 10 times that of α-tocopherol [164]. Its antioxidant actions are attributed mainly to its chemical structure. Lycopene has been confirmed as able to scavenge ONOO^−^, nitrogen dioxide as well as thiol and sulphonyl radicals [61,165]. Recently, Sheikholeslami et al. [90] observed that the addition of lycopene to an extender increased motility and viability and reduced the LPO of dog sperm compared to the control group (Table 3).

## 6. Treatments with Antioxidants in Livestock Species

**Figure 1:**
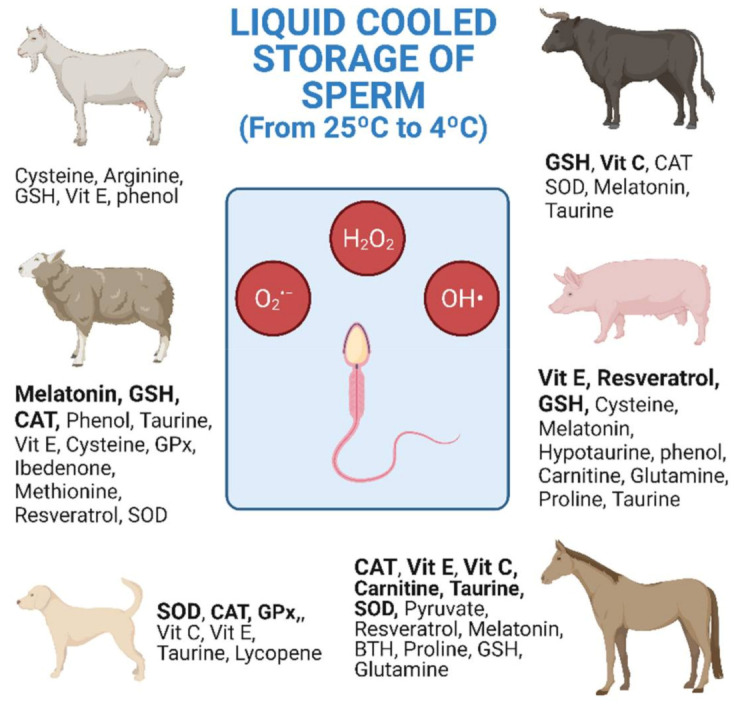
Antioxidants used in cooled semen storage by species. Research studies listed in Table 1, Table 2 and Table 3 collected together and distributed by animal species. Bold: 2 or more different research studies carried out (both alone and in combination) (Created with BioRender.com).

The research studies shown in Table 1, Table 2 and Table 3 are collected and distributed by animal species in Figure 1. The figure shows the antioxidants used, as well as the number of investigations carried out according to the animal species. Equine and ovine, closely followed by porcine, are the animal species for which the greatest number of studies of antioxidants in cooled semen have been carried out (Figure 1).

In general, cooled semen has a greater longevity in the female tract than frozen semen [166]. This means that AI with frozen semen must be more precise for both the deposition place and ovulation time [166]. In sheep, the explanation for the great interest in improving cooled storage may be due to various reasons including the difficulty of carrying out post-cervical AI and the low longevity of the refrigerated semen. The cervix of the sheep is intricate and post-cervical AI is more difficult than with other species such as cattle or goats. In ovine, the most widely investigated antioxidants are CAT, GSH and melatonin, the latter giving the best results with refrigerated semen (Figure 1 and Table 3 [144,145,147]). In equine, AI with refrigerated semen is easier and achieves higher pregnancy rates than when using frozen–thawed semen [167]. Moreover, semen doses from valuable stallions reach high prices in the market [168]. In stallions, CAT, SOD, GSH, vitamin E, carnitine and taurine are the most investigated antioxidants. However, only the amino acids showed repeated beneficial results on sperm motility with refrigerated semen (Figure 1 and Table 3 [125,126,130,132]). In pigs, although semen freezing was developed many years ago, AI with refrigerated semen is still by far the most commonly used method [169]. In this species, the most studied antioxidants are GSH, vitamin E and, more recently, resveratrol, but without achieving clear conclusive results for cooled semen storage.

## 7. Final Considerations and Conclusions

Although antioxidant supplementation in semen extenders was developed many years ago, the problem of OS in cooled storage of semen has not been fully resolved. The effects of antioxidant supplementation have continued to be investigated very actively in recent years. In fact, many of the works cited in this review were published after 2015; the number of studies and antioxidant used varies depending on the animal species. Besides biological differences in the sperm of different species, one of the main variables could be the antioxidant concentration used. It is, therefore, crucial to adjust the appropriate antioxidant concentration. However, this “optimal” concentration could vary depending on various factors, such as the OS state of the semen sample. For example, Jofré et al. [88] observed that antioxidant supplementation with a phenol extract had a detrimental effect on boar spermatozoa, while this antioxidant improved the semen quality parameters in samples previously treated with H_2_O_2_. In another study, the optimum concentration of resveratrol was 50 µM at 17 °C but, if the boar semen sample was rapidly refrigerated at 5 °C, the optimum concentration was 150 µM [154]. Care should be taken to avoid excessive antioxidant concentrations, as this may have a detrimental effect on sperm quality (Table 1, Table 2 and Table 3). We think it would be important to evaluate oxidation level of sperm sample in particular conditions (storage temperature, sperm concentration, extender, storage time, etc.) before adding any antioxidant substance. Some studies finding no beneficial effect of antioxidants did not evaluate ROS levels or direct effects (e.g., lipid peroxidation) in the sperm samples previous to antioxidant supplementation [94,95,106,122]. Although in most studies of semen refrigerated storage, either an increase in ROS levels or its direct effect were detected, these effects could not happen [101,105].

The results of antioxidant supplementation also varied according to storage time. Beneficial effects were normally observed during long storage periods, while no clear effects were observed during the first hours of storage [79,87,88,94,104]. Prolonged cooled storage may provoke a higher OS state of the semen sample than short storage. Moreover, heterogeneity in semen preparation and refrigerated protocols in different species, even intra-species, could affect results such as, for example, the presence of SP in cooled samples. Different works indicated that sample washing tended to reduce sperm motility [3,170,171], although it depended on duration and g-force of centrifugation. Furthermore, the number of studies about the effects of antioxidant supplementation of refrigerated semen on in vivo fertility is low when compared to in vitro studies. Most of the former studies did not observe beneficial effects on in vivo fertility of antioxidant supplementation in cooled semen storage [11,82,118,149,152]. However, some researchers observed beneficial effects on in vivo fertility [82] or prolificacy [11]. Once again, this effect may depend on the storage conditions, as beneficial effects have been reported for CAT supplementation on in vivo fertility when the samples were stored at ambient temperature, but not when they were stored at 5 °C [82].

As other technologies advance, new substances or strategies have been proposed, such as the use of nanoparticles with scavenger activity to avoid OS [172], or more in-depth studies of promising natural (in general, phenols [149,173]) or synthetic substances [89], of which few have been studied to date in the cooled storage of semen. More research is needed to adjust antioxidant concentrations and study new substances with antioxidant effects.

## Figures and Tables

**Table 3:** Effects of non-enzymatic antioxidants (vitamins, phenols, indoles and other types) supplementation in liquid cooled storage on sperm quality parameters.

Antioxidant	T	Concentr	Opt	A/C	Temp	Time	Species	In Vitro Effects	Ref.
Vit C + GSH		0.5–2 mg/mL + 1 mM		C	5 °C	24 h	bovine	No effect on studied parameters.	[94]
BHT		0.5–2 mM		A	5 °C	72 h	equine	Detrimental effect on motility.	[106]
Idebenone	Bq	1–8 µM		A	5 °C	72 h	ovine	Increased motility, progressivity, viability and T-AOC.	[89]
Lycopene	Crt	250–750 µg/ml	500 µg/ml	A	5 °C	72 h	canine	Increased motility, progressivity, viability and T-AOC and reduced MDA.	[90]
Melatonin	Idl	1 µM		A	17 °C	7 d	porcine	Detrimental effect on sperm motility and viability on day 7.	[143]
Melatonin	Idl	1–4 mM	3 mM	A	4 °C	30 h	bovine	Increased motility and decreased dead or abnormal spermatozoa, and acrosomal abnormalities.	[83]
Melatonin	Idl	0.3 mM		A	4 °C	48 h	ovine	Increased motility, viability and T-AOC. Reduced MDA.	[144]
Melatonin	Idl	0.05–0.4 mM	0.1 mM	A	4 °C	5 d	ovine	Improved motility, viability, mitochondrial activity and T-AOC and reduced MDA. Toxic at High concentrations.	[145]
Melatonin	Idl	1–2 mM	1.5 mM	A	5 °C	48 h	equine	Increased motility and viability. Reduced MDA	[93]
Melatonin	Idl	1 µM		A	5 °C	6 h	equine	No effect on sperm motility, viability or ROS. Increased mitochondrial activity and intact acrosome.	[146]
Melatonin	Idl	0.1–3 mM	1 mM	A	5 °C	48 h	ovine	Improved progressive motility.	[147]
Melatonin	Idl	0.5–1.5 mM	1 mM	A	5 °C	24 h	rabbit	Increased sperm motility, viability and reduced DNA fragmentation.	[148]
HT and DHPG	Phl	5–100 µg/mL for each one		A/C	15 °C	96 h	ovine	No effect in general, only affected VLC.	[149]
HT and DHPG	Phl	5–100 µg/mL for each one		A/C	5 °C	96 h	ovine	Only affected some kinetic parameters of motility.	[149]
polyphenol- murtilla	Phl	0.0315 μg GAE mL		A	17 °C	7 d	porcine	Increased motility, viability. Reduced ROS production.	[88]
procyanidin extract	Phl	10–70 mg/L	30 mg/L	A	5 °C	120 h	caprine	Increased motility, viability, acrosome integrity, mitochondrial activity and T-AOC. Reduced MDA.	[11]
Resveratrol	Phl	10–80 µM		A	10 °C/4 °C	24 h	equine	Detrimental effects at high concentration.	[150]
Resveratrol	Phl	200–400 uM		A	5 °C	168 h	ovine	Improved motility, kinematic parameters and in vitro fertility, antioxidant activities and reduced oxidative stress	[151]
Resveratrol	Phl	0.01–1 mM		A	17 °C	72 h	porcine	No positive effect on motility or kinetics, viability. Reduced SOD. Toxic at high concentrations.	[152]
Resveratrol	Phl	10–100 µM		A	17 °C	4–7 d	porcine	No positive effect on motility or kinetics. Toxic at high concentrations.	[153]
Resveratrol	Phl	25–150 uM	50 uM	A	17 °C	5 d	porcine	Increased sperm motility, membrane integrity and mitochondrial activity and T-AOC levels. Decreased ROS and MDA.	[154]
Quercetin	Phl	25–200 µM		A	5 °C	96 h	rabbit	No effect on motility, kinetics or DNA fragmentation. Reduced intracellular H_2_O_2_. Toxic at high concentrations.	[136]
Quercetin	Phl	25–200 µM		A	15 °C	96 h	rabbit	No effect on motility, kinetics or DNA fragmentation. Reduced intracellular H_2_O_2_. Toxic at high concentrations.	[136]
Vit C	Vit	0.5 mM		A	4 °C	72 h	canine	No effect on sperm motility, increased sperm viability.	[105]
Vit C	Vit	0.5–2 mg/mL		A	5 °C	24 h	bovine	No effect.	[94]
Vit C	Vit	0.45–0.9 mg/mL		A	5 °C	72 h	equine	Increased sperm viability, at high concentrations reduced motility.	[95]
Vit C	Vit	1–4 mM		A	5 °C	72 h	equine	No significant effect.	[106]
Vit E	Vit	0.1 mM		A	4 °C	72 h	canine	Reduced total ROS, increased motility and viability.	[105]
Vit E: trolox	Vit	2 mM		A	5 °C	72 h	equine	No effect on motility.	[106]
Vit E: Trolox	Vit	0.2–5 mM		A	5 °C/15 °C	96 h	ovine	None or detrimental effect on motility and viability (improved mitochondrial activity at 5ºC); depending on extender.	[118]
Vit E: α-tocopherol	Vit	3 mM		A	5 °C	72 h	caprine	Increased progressive motility and viability, reduced lipid peroxidation.	[113]
Vit E: α-tocopherol	Vit	5–10 mM		A	5 °C	48 h	equine	No effect on motility or viability or MDA.	[140]
Vit E: α-tocopherol	Vit	1–4 mM		A	5 °C	72 h	equine	No effect on motility.	[106]
Vit E: α-tocopherol	Vit	200 µM		A	17–15 °C	72 h	porcine	No effect on motility and host (in non-dialyzed samples).	[141]
Vit E: α-tocopherol	Vit	0.2 mg/mL		A	19 °C	5 d	porcine	Improved sperm viability.	[142]

HT: dihydroxyphenylethanol; DHPG: dihydroxyphenylglycol; BHT: butylated hydroxytoluene; T: type; Bq: benzoquinone; Crt: carotenoid; Idl: indole; Phl: phenol; Vit: vitamin; Opt: optimum concentration; A: alone; C: combination; Time: time of cooled storage; d: days; h: hours; Temp: temperature; SOD: superoxide dismutase; ROS: reactive oxygen species; VLC: curvilinear velocity; MDA: malondialdehyde; T-AOC: Total antioxidant capacity; Ref.: reference.
